# Interpretable Feature Selection and Hybrid Deep Learning Models for Depressive Symptoms Prediction from Wearable Device Data

**DOI:** 10.1007/s10916-026-02354-9

**Published:** 2026-03-03

**Authors:** Jaehoon Ko, Somin Oh, Doljinsuren Enkhbayar, Jin-kyung Lee, Moo-Kwon Chung, Taeksoo Shin, Min-Hyuk Kim, Hyo-Sang Lim, Erdenebayar Urtnasan, Jaehong Key

**Affiliations:** 1 Department of Biomedical Engineering, Yonsei University, Wonju, Republic of Korea; 2 Institute for Poverty Alleviation and International Development, Yonsei University, Wonju, Republic of Korea; 3 Department of Global Public Administration, Yonsei University, Wonju, Republic of Korea; 4 Division of Business Administration, Yonsei University, Wonju, Republic of Korea; 5 Department of Psychiatry, Yonsei University Wonju College of Medicine, Wonju, Republic of Korea; 6 Division of Software, Yonsei University, Wonju, Republic of Korea; 7 Division of AI Semiconductor, Yonsei University, Wonju, Republic of Korea

**Keywords:** Wearable device, Deep Learning, SHAP, LIME, Depression

## Abstract

Early detection and prediction of Depressive Symptoms is essential for improving mental health outcomes. This study proposes a hybrid deep learning and machine learning framework that utilizes tabular data collected from wearable devices, including sleep patterns, physical activity, and health-related indicators. Three ensemble learning models were used to identify influential predictors through the application of explainable artificial intelligence techniques such as SHAP and LIME. Based on the selected important features, two hybrid models were developed combining 1D-Convolutional Neural Network and Multi-layer Perceptron with LightGBM. The experimental results showed that models trained on selected features consistently outperformed those using the full feature set. The highest classification accuracy, 93.43%, was achieved by the multilayer perceptron model with LightGBM when trained on features selected by XGBoost. SHAP analysis highlighted the importance of features such as night sleep duration, age, and income responsibility, while LIME provided sample specific explanations that enhanced local interpretability. This framework enhances both the predictive performance and interpretability of depression prediction models and demonstrates the potential of wearable-derived behavior and physiological features as practical biomarkers for personalized risk assessment.

## Introduction

In the past, medical efforts primarily focused on treating infectious diseases such as the plague or acute physical illnesses. However, in modern society, increasing emphasis is being placed on the prevention and treatment of mental disorders, which are directly linked to the quality of life [1]. Mental health is not merely associated with psychological stability or subjective well-being but is also closely related to critical outcomes such as suicide rate, highlighting its significance as a public health concern [2, 3].

Among various mental disorders, Major Depressive Disorder (MDD) is one of the most common and serious conditions. Particularly in the aftermath of the COVID-19 pandemic, the global prevalence of depression has surged, with certain countries experiencing a disproportionately sharp increase [4–6]. This trend underscores the urgent need to address depression as a critical health issue, emphasizing the importance of early diagnosis and preventive strategies. To achieve this, the identification of accurate bio-makers and systematic analysis of features closely associated with depression are essential. Currently clinical diagnosis of depression largely relies on psychiatric consultations or self-report questionnaires. However, these approaches often depend heavily on patient’s subjective recall of their psychological state, past stress experiences, and environmental factors, thereby lacking consistency and objectivity [7]. Moreover, diagnosis and treatment are only possible if the individual voluntarily seeks help, which imposes another limitation. Given these challenges, the development of an objective and automated system for early detection and prediction of depression has become increasingly important [8–10].

In this context, Artificial intelligence (AI) has emerged as a promising solution. AI technologies can process and learn from large scale data, and environmental data offers great potential to supplement or even replace traditional, subjective diagnostic methods. Such systems can facilitate the establishment of a more quantitative, proactive, and predictive mental health management framework [11, 12].

In this study, data were collected using accessible devices such as smartwatches and smartphone applications, enabling real world data acquisition beyond the constraints of clinical settings. This approach allowed for the inclusion of practical and reliable variables measured in everyday life, rather than controlled clinical environments, in order to develop a more realistic and robust depression prediction model. Furthermore, we analyzed and visualized the relationships between the collected features and depression status to validate their relevance to mental health. To this end, machine learning algorithms were employed to analyze key features, and deep learning models were applied to enhance predictive performance. In addition, explainable AI (XAI) techniques were incorporated to interpret the decision-making process of the models and to visualize the influence of individual features. The goal of this study is to improve the feasibility of early depression diagnosis and to contribute to the foundation of a more objective and predictive mental health management system.

## Methods

**Fig. 1:**
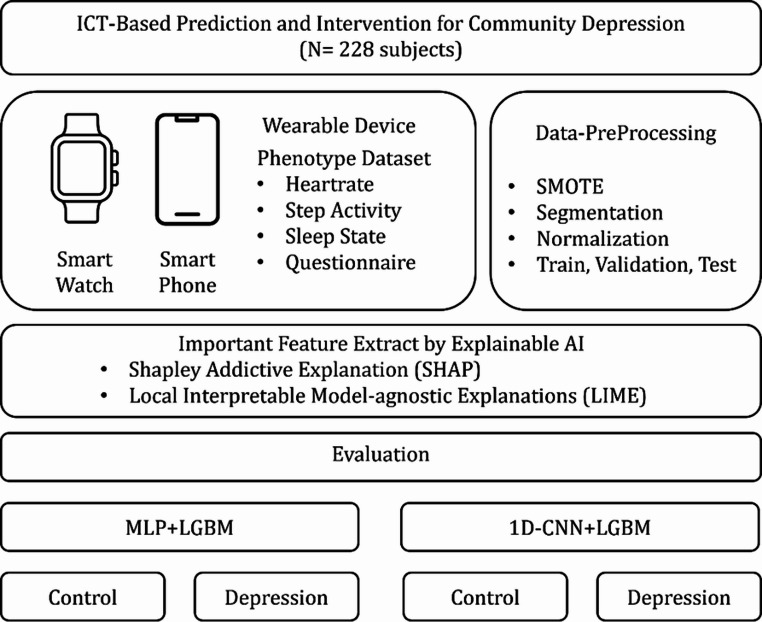
Workflow of the proposed ICT-based depression prediction model using wearable device data from 287 subjects. Key features were extracted via SHAP and LIME, and hybrid models (MLP+LGBM, 1D-CNN+LGBM) were trained to classify control depressed groups

This study aims to develop a depression risk assessment model by preprocessing and refining life log data collected from a total of 287 individuals, and by identifying key features that are directly associated with depressive symptoms. To achieve this, three ensemble learning models were employed, and explainable AI (XAI) tools such as SHAP and LIME were utilized to enhance the interpretability and reliability of the feature analysis. Furthermore, to gain deeper insights into how the extracted features contribute to the model’s predictive performance, a deep learning–based architecture was implemented and analyzed. The overall research flow and analytic framework are visually summarized in the schematic diagram presented in Fig. 1.

### Participants

This study was conducted using data collected from a community-based cohort study (IRB No.1041849-202401-SN-020-11). The target population consisted of middle-aged and older adults aged 55 to 85 years residing in the city of Wonju, South Korea. Between December 2020 and April 2023, a total of 685 participants were enrolled in the study. Among them data from 228 individuals who continuously provided data over the three-year period were included in the analysis. Each participant was provided with Samsung Galaxy 2 and was instructed to wear it continuously in their daily lives, allowing for passive data collection. Physiological indicators such as step activity, heartrate, and sleep states were collected through the smartwatch, while a synchronized smartphone application captured digital behavior data including the number of text messages sent, call logs, smartphone usage patterns, and message content. In addition, participants completed a series of Ecological Momentary Assessments (EMA), including a daily mood questionnaire, weekly reports of significant stress events, and monthly depression assessments. Furthermore, structured survey data were collected to assess each participant’s personal health conditions, employment and income status, and family history of physical and mental illnesses, enabling a comprehensive profiling of individual lifestyle and socioeconomic background.

To assess depressive symptoms, the widely used self-report instrument PHQ-9 (Patient Health Questionnaire) was administered. The PHQ-9 consists of nine items, each scored on a scale from 0 to 3 according to four response options: not at all, several days, more than half days, and nearly every day. The total score ranges from 0 to 27, with higher scores indicating greater depressive symptoms severity. In this study, participants scoring between 0 and 4 were classified as the non-depressive group, while those scoring 5 or higher were classified as the depressive group [13]. Based on this criterion, out of 228 participants, 129 were categorized as non-depressed and 99 as potentially depressed. Table 1 summarizes the demographic and health-related characteristics of the participants. Approximately 58% of the participants were female, and about 68% were non-smokers. Most of participants were in their 60s, accounting for roughly 50% of the total subject. Regarding chronic disease history, 93% of participants had a history of cerebrovascular disease, and 86% had experienced or been diagnosed with cancer, indicating that a large portion of the participants had one or more existing health conditions. In terms of household income, the 31% proportion of participants reported monthly earnings in the range of 2 to 3.5 million KRW. These findings provided a valuable foundation for developing predictive models for mental health status using real-life, multidimensional physiological and behavior data, supporting the potential for early detection and proactive mental health management.

**Table 1:** Sociodemographic, clinical, and economic characteristics of participants according to depression status

Variables		CTL (*n* = 129)	DS (*n* = 99)	Total
Sex				
	Male	58 (25.44%)	37 (16.23%)	95
	Female	71 (31.14%)	62 (27.19%)	133
Age group				
	< 60	11 (4.82%)	7 (3.07%)	18
	60 ~ 70	64 (28.07%)	51 (22.37%)	115
	70 ~ 80	47 (20.61%)	33 (14.47%)	80
	80 ~ 90	7 (3.07%)	8 (3.51%)	15
Smoking Status				
	Non-Smoker	83 (26.40%)	71 (31.14%)	154
	Former Smoker	41 (17.98%)	25 (10.96%)	66
	Current Smoker	5 (2.19%)	3 (1.32%)	8
Disease Status				
	Hypertension	73 (32.02%)	70 (30.70%)	143
	Hyperlipidemia	70 (30.70%)	49 (21.49%)	119
	Diabetes	105 (46.05%)	81 (35.53%)	186
	Heart disease	110 (48.25%)	80 (35.09%)	190
	Cerebrovascular disease	120 (52.63%)	93 (40.79%)	213
	Cancer	113 (49.56%)	84 (36.84%)	197
Household Income				
	~ 2 M KRW	27 (11.84%)	28 (12.28%)	55
	2 ~ 3.5 M KRW	37 (17.11%)	34 (14.91%)	71
	3.5 ~ 5 M KRW	40 (17.54%)	23 (10.09%)	63
	5 M KRW	25 (10.96%)	14 (6.14%)	39

Note: CTL=Control group, DS=Depressive Symptoms group, M=Million, KRW=Korean Won.

## Data Preprocessing

In this study, a total of 61 features were utilized for model training, including key variables such as step count, heartrate, sleep status, and responses to the questionnaire. Given the significant differences in value ranges among these variables, normalization was deemed necessary to ensure the stability and performance of the machine learning algorithms. To address this, the MinMaxScaler function from the Scikit-Learn library was applied to re-scale the data. MinMaxScaler normalizes each feature by linearly transforming its values to a fixed range between zero and one, based on the feature’s minimum and maximum values. This prevents certain features from disproportionately influencing the learning process and helps improve both convergence speed and predictive performance of the models [14–16]. The normalization process was conducted following Eq. (1).

1 $$X_{scaled}=\frac{X-X_{min}}{X_{max}-X_{min}}$$

A total of 287 participants were included in the dataset, and to augment the number of training samples, data from each participant were segmented into monthly units. Rather than treating the dataset as continuous time-series signals, monthly average values of physiological indicators were computed and paired with the corresponding PHQ-9 scores, resulting in one sample per month. Samples with missing values were excluded from the analysis. In addition, to reduce temporal dependency between consecutive observations, samples from adjacent months were excluded as a conservative preprocessing step. This exclusion was applied as a conservative preprocessing choice, informed by prior observations that behavioral and physiological changes associated with depressive symptoms may extend beyond a single assessment point and exhibit short-term temporal persistence [17, 18]. As a result, a total of 1,582 monthly samples were obtained, comprising 1,105 control samples and 477 samples with elevated depressive symptoms. To further characterize the composition of the study cohort, participants were descriptively categorized according to PHQ-9 score ranges reflecting different levels of depressive symptoms severity (< 5, 5–9, 10–14, 15–19, and ≥ 20). The distribution of samples across these severity levels is summarized in Table 2. This descriptive analysis was performed to provide transparency regarding the severity profile of the dataset and to clarify that the depressive symptoms group encompassed a spectrum ranging from mild to severe symptoms levels.

**Table 2:** Sample Counts of Depressive Symptoms Severity Levels Based on PHQ-9 score ranges

Depressive Symptom Severity	PHQ-9 score range	Male	Female	Total
Minimal	< 5	476	600	1,076
Mild	5 ~ 9	130	209	339
Moderate	10 ~ 14	37	65	102
Moderately Severe	15 ~ 19	11	27	38
Severe	$\:\ge\:$20	7	20	27
Total		661	921	1,582

To mitigate potential model bias toward the majority class and to improve sensitivity to minority class predictions, we applied the Synthetic Minority Over-sampling Technique (SMOTE) with an oversampling ratio of 0.5 to maintain class balance without duplicating samples thereby reducing the risk of overfitting often associated with simple oversampling. SMOTE generates synthetic minority class samples by interpolating between existing data points in the feature space, effectively augmenting the dataset while preserving the original distribution [19–21]. Specifically, the number of depressive symptoms samples in the training set increased from 477 to 553 through SMOTE based augmentation, resulting in a total of 1,658 training samples. The validation and test sets remained unchanged to ensure an unbiased performance evaluation. The detailed composition of each dataset subset is summarized in Table 3.

**Table 3:** Distribution of Samples in the Training, Validation, and Test Sets After Applying SMOTE to Balance the Training Set at a 0.5 Oversampling Ratio

Training	CTL	DS	Total
	663	361	1,024
Validation	221	96	317
Testing	221	96	317
Total	1,105	553	1,658

Subsequently, the entire dataset was split into 60% for training, 20% for validation, and 20% for testing. A Stratified K-Fold cross validation strategy was adopted to ensure that the class distribution was preserved in each fold [22, 23]. The training set was used for model learning, the validation set was used for hyperparameter tuning and model selection, and the test set served as an independent evaluation dataset to access final model performance.

## Machine Learning Models

Machine learning algorithms are known for their strong predictive performance when applied to structured tabular data, and they offer the added advantage of seamless integration with XAI techniques [24, 25]. In this study, we leveraged these strengths to perform feature-level analyses using three representative machine learning models commonly employed in structured data domains. Rather than conducting a comparative evaluation of model performance, our primary objective was to identify and interpret the key predictive features contributing to depression classification through explainable learning methods. Among various machine learning paradigms, tree-based ensemble algorithms have demonstrated notable effectiveness in handling structured data and were therefore selected as the primary models in our analysis.

Ensemble learning is a technique that combines multiple base learners to construct a more robust and generalized predictive model. This approach is particularly effective when employed with high-variance models such as decision trees, as it helps mitigate overfitting and improves generalization performance [26, 27]. The performance advantage of ensemble models stems from the diversity and independence among base learners, which allows the system to aggregate complementary error patterns and enhance overall predictive reliability. Ensemble methods are generally categorized into bagging, boosting, and stacking, depending on their integration strategies. In this study, we focused on bagging and boosting approaches for the purpose of feature importance analysis [28]. Bagging trains multiple instances of the same model on different subsets of the training data typically using bootstrap sampling to reduce variance. The final prediction is obtained through majority voting in classification tasks or averaging in regression tasks. A representative bagging-based algorithm, Random Forest, was used to derive global feature importance rankings in our study. In contrast, boosting builds an ensemble sequentially, where each new learner is trained to correct the errors of the previous ones. This sequential focus on harder-to-classify instances leads to progressively refined decision boundaries. The final output is generated by aggregating the predictions of all learners using a weighted voting scheme. In this context, we employed two widely used boosting algorithms XGBoost and LightGBM with the specific aim of extracting interpretable insights regarding the relative contributions of individual input features [29, 30].

## Deep Learning Models

With the recent advancement of artificial intelligence technologies, deep learning has emerged as a core technique for solving complex pattern recognition and prediction tasks. Among various deep learning architectures, Multi-Layer Perceptron (MLP) and Convolutional Neural Network (CNN) have demonstrated remarkable capabilities in capturing nonlinear relationships among input variables and extracting meaningful patterns from locally structured data [31]. While originally developed for image and signal processing, these models have also been effectively applied to structured numerical data, commonly referred to as tabular datasets [32–34]. However, using a single deep learning architecture alone may not be sufficient to capture the diverse and complex relationships embedded within high-dimensional tabular data. This approach aims to leverage the complementary strengths of deep learning and ensemble-based machine learning for enhanced predictive performance.

In the proposed hybrid model, tabular input data is fed into either an MLP or a 1D-CNN architecture, depending on the experimental setting. The MLP module consists of three fully connected dense layers with 128, 64, and 32 hidden units, respectively, each followed by ReLU activation and dropout regularization to prevent overfitting.

**Fig. 2:**
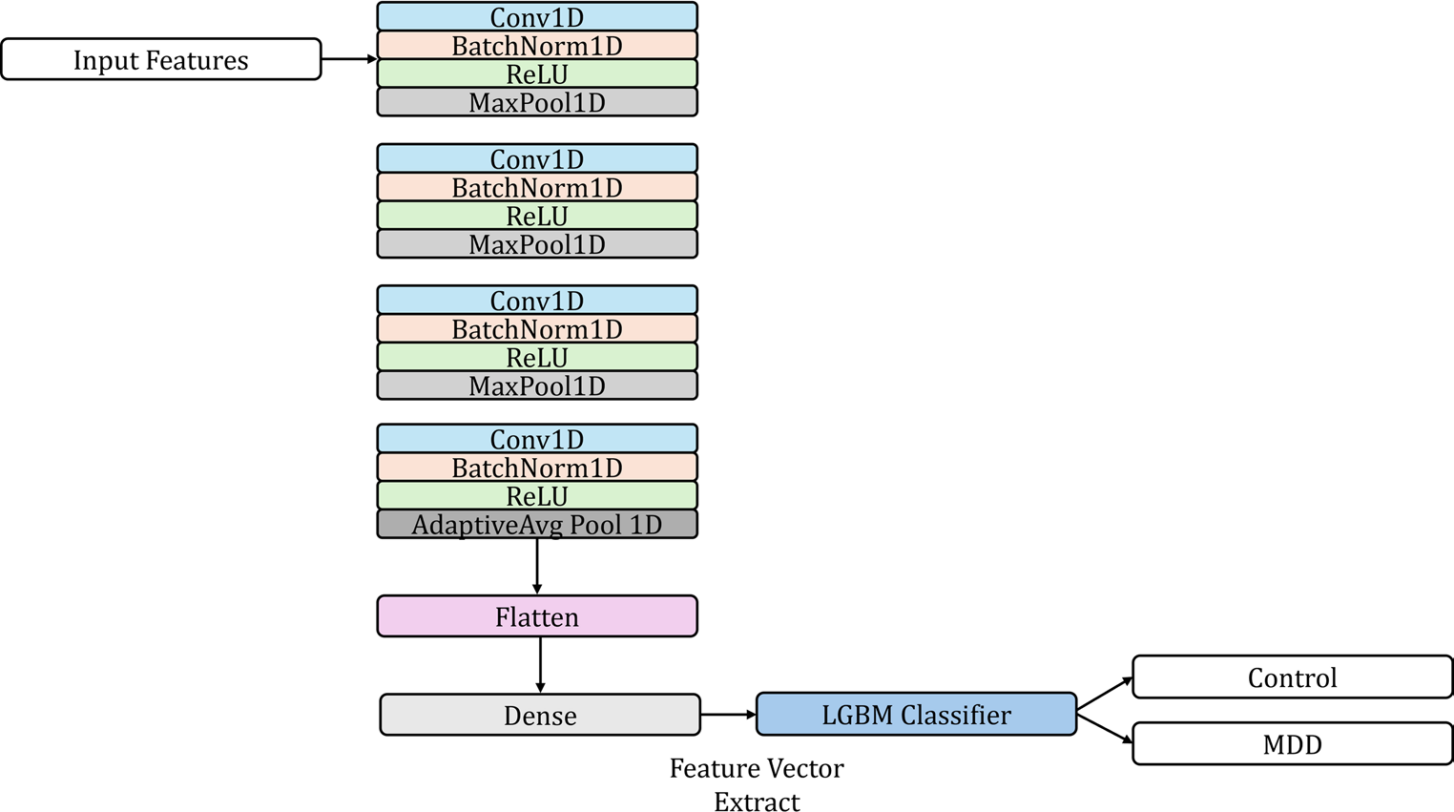
Architecture of the proposed hybrid model. The input tabular data is processed through a 1D-CNN network consisting of four convolutional blocks, each followed by batch normalization, ReLU activation, and pooling layers. The final feature embedding is obtained after a fully connected layer and passed to a LightGBM classifier for final prediction

**Fig. 3:**
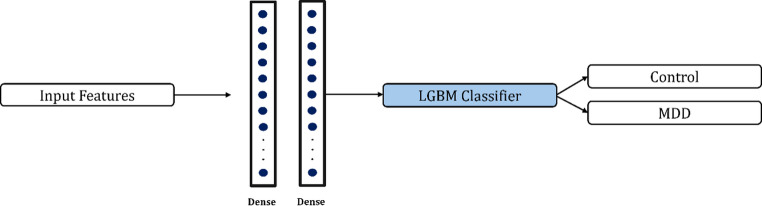
Architecture of the proposed hybrid model based on MLP and LightGBM. The input tabular features are passed through four fully connected layers with dropout regularization to learn high-level feature representations. The resulting embedding is used as the input for a LightGBM classifier, which performs the final binary classification of depression vs. control

The output of the final hidden layer is treated as a high-level feature embedding vector extracted by the MLP. On the other hand, the 1D-CNN module treats the tabular features as a single-channel input and applies a series of four convolutional layers with 16, 32, 64, and 128 filters, respectively. Each convolutional layer uses a kernel size of 3 and is followed by batch normalization, ReLU activation, and max pooling to progressively extract local patterns while reducing dimensionality. After convolutional operations, an adaptive average pooling layer and a fully connected layer with 64 units are employed to generate the final feature vector from the CNN branch. For both architectures, the extracted feature vectors are subsequently converted into numpy arrays and used as inputs for a LightGBM classifier. During training, the deep learning modules act solely as feature extractors, while LightGBM performs the final classification based on the learned representations. This hybrid architecture strategically combines the expressive power of deep neural networks for feature extraction with the robust predictive capability of gradient boosting methods, aiming to achieve improved performance on structured tabular datasets. Figures 2 and 3 illustrate the architectural diagrams of the CNN-based and MLP-based hybrid models employed in this study.

## Hyperparameter Optimization

The performance of machine learning and deep learning models is influenced not only by their structural design but also by the configuration of various hyperparameters that govern the training process. Hyperparameters are values that must be specified prior to training and are not learned directly from the data [35, 36]. In deep learning, common hyperparameters include the learning rate, batch size, and dropout rate, while in tree-based machine learning models, parameters such as max depth and n-estimators are particularly critical. The choice of hyperparameters significantly affects factors such as convergence speed, generalization ability, and overfitting prevention. Therefore, systematic hyperparameter optimization is essential to achieve optimal model performance. In this study we employed the Grid Search technique, which explores predefined hyperparameter space and selects the best performing configuration based on validation metrics. The hyperparameter ranges and the best combinations for each machine learning model are summarized in Table 4. For training deep learning models, the Adam optimizer was used with an initial learning rate set to 0.001. To ensure stable learning and efficient convergence, the batch size was set to 64, and the model was trained for a total of 40 epochs. The loss function used was the Binary Cross Entropy with Logits selected to optimize feature representation in a regression-based context. The detailed hyperparameter settings applied to the deep learning models are presented in Table 5.

**Table 4:** Overview of the hyperparameter search space and the corresponding optimal values for the machine learning models used in this study. The hyperparameters were tuned using Grid Search, and the best combination was selected based on cross-validated performance metrics

Model	Hyperparameter space	Best Hyperparameter
Random Forest	n_estimators : 3–9 max_depth : 1–5 min_samples_split : 2–5 min_samples_leaf : 1–2	n_estimators : 3 max_depth : 5 min_samples_split : 2 min_samples_leaf : 1
XGBoost	n_estimators : 3–9 max_depth : 1–5 min_samples_split : 2–5 min_samples_leaf: 1–2	n_estimators : 5 max_depth : 5 min_samples_split : 2, min_samples_leaf : 2
LightGBM	n_estimators : 3–9 max_depth : 1–5 min_samples_split : 2–5 min_samples_leaf: 1–2	n_estimators : 3 max_depth : 5 min_samples_split : 2 min_samples_leaf: 2

**Table 5:** Summary of the hyperparameter configuration used in the hybrid model. This table presents key training-related parameters including the optimizer type, learning rate, batch size, number of epochs, and loss function

Hyperparameter	1D-CNN + LGBM	MLP + LGBM
Batch Size	64	64
Loss	Binary Cross Entropy with Logits	Binary Cross Entropy with Logits
Optimizer	Adam	Adam
Learning Rate	1e-3	1e-3
Epochs	40	40

### Explainable AI

In recent years, there has been a growing interest in XAI (Explainable Artificial Intelligence) as the need to interpret and understand the decision-making processes of complex AI models has become increasingly important. While deep learning-based models often demonstrate high predictive performance, they are typically regarded as black-box systems, making it difficult to clearly identify the rationale behind specific predictions. This limitation has highlighted the importance of techniques that can provide more intuitive and quantitative interpretations of model outputs. XAI aims to enhance the transparency and reliability of AI systems by offering explanations of why a particular input leads to a given prediction, and identifying which features have the most significant influence on the model’s decision [37]. Among the various XAI techniques, SHAP (Shapley Addictive Explanations) and LIME (Local Interpretable Model-agnostic Explanations) are widely used. These methods quantitatively assess the contribution of each input variable to the final prediction or use local surrogate models to approximate and interpret the behavior of complex models in a human understandable manner. Such explanation-based analysis extends beyond predictive accuracy by enabling researchers to validate and interpret the underlying reasoning of the model, thereby enhancing both its practical utility and interpretability [38, 39]. In this study, SHAP and LIME are applied to quantitatively analyze the relationship between life log-based behavior data and depression, and to compare the results with established clinical knowledge. Through this process, the study aims to explore the potential of XAI in identifying interpretable digital biomarkers related to depressive symptoms.

## Result

In this study, we applied three ensemble learning models Random Forest, XGBoost, and LightGBM to identify and analyze the most important features associated with depression. To improve the interpretability of the feature selection process, we employed XAI techniques, including SHAP and LIME, which enabled the quantitative assessment of each feature’s contribution to model predictions. Through this process, we identified candidate biomarkers potentially associated with depressive symptoms. Using these selected features, we developed two deep learning-based hybrid models MLP + LGBM and 1D-CNN + LGBM to predict depression outcomes and compared their performance with models trained on the full set of features. All Models were implemented using the PyTorch framework. The dataset was divided into 60% training, 20% testing, and 20% validation sets, with model training and evaluation conducted through 5-fold cross validation to ensure robustness. All experiments were performed on a workstation equipped with an Intel Core i7-14700k CPU, 32GB of RAM, and NVIDIA RTX 3060 GPU.

## SHAP Analysis

SHAP (SHapley Additive eXplanations) is one of the representative XAI techniques used to interpret the prediction results of machine learning models. It has the advantage of quantifying and visualizing how much and in which direction each feature contributes to the model’s prediction outcome. The SHAP value, obtained through this method, serves as a key component in decomposing the prediction output, indicating the extent to which each feature influences the model’s overall prediction value, as described in Eq. (2).

2 $$f(x)=E\left(\left[f\left(x\right)\right]\right)+\sum_{i=1}^n\phi_i$$

**Fig. 4:**
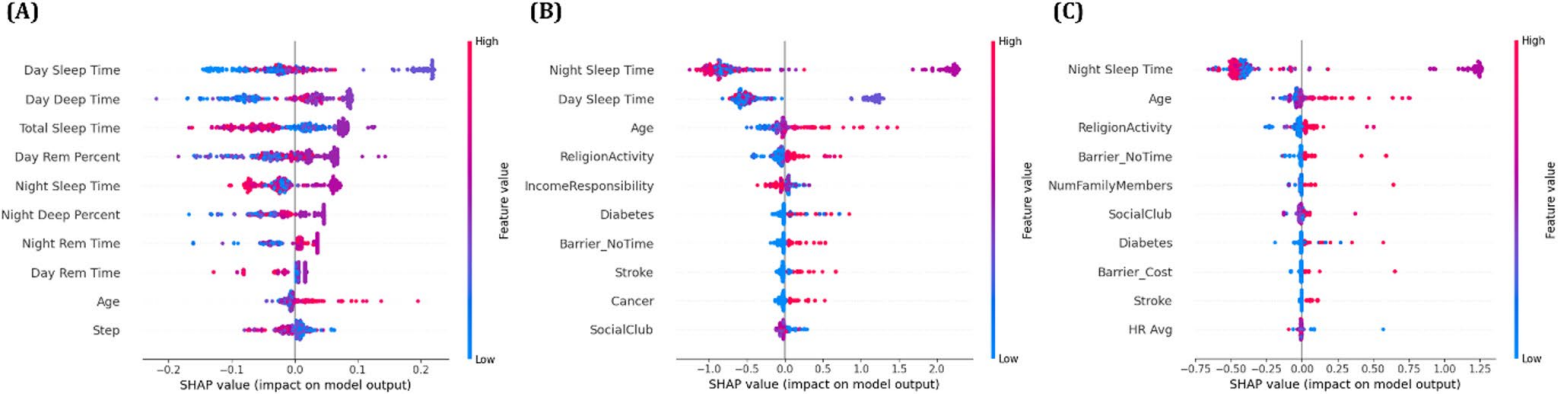
SHAP beeswarm plots for depression prediction models: (**A**) Random Forest, (**B**) XGBoost, and (**C**) LightGBM. Each plot shows the global feature importance and the direction of influence of features on the model’s prediction. Colors indicate the original feature values, and features are sorted by their mean absolute SHAP values

**Fig. 5:**
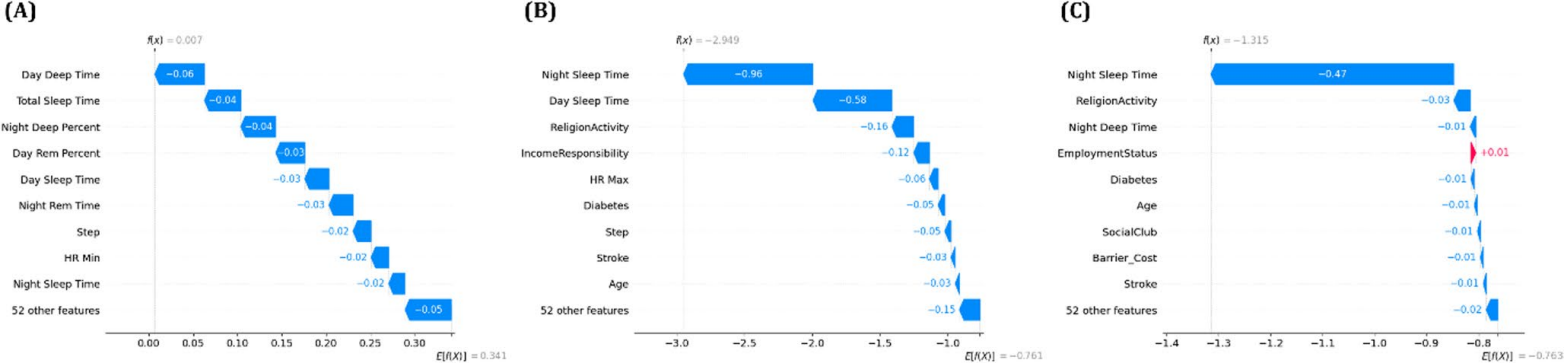
SHAP waterfall plots show the contribution of each feature to the final prediction for a representative individual. (**a**) Random Forest, (**b**) XGBoost, and (**c**) LightGBM. Each plot illustrates how the model’s prediction is built from the base value by adding the SHAP values of individual features. Features pushing the prediction higher are shown in red, while those lowering it are shown in blue

In this equation, *f(x)* represents the model’s prediction for an input sample *x*, and *E[f(x)]* denotes the average prediction across the entire dataset, serving as the baseline value. *ϕ*_*i*_ refers to the contribution of feature *i* to the model’s prediction, and by comparing these contributions, the relative importance of each feature to the final prediction can be systematically accessed. Therefore, SHAP values enable the decomposition of complex decision-making processes within the model, allowing the identification of which features exert a stronger or weaker influence on the output prediction. Based on this interpretability, this study employed SHAP-based visualization techniques, namely the beeswarm plot and waterfall plot, to analyze the key features involved in the depression prediction models. The beeswarm plot summarizes SHAP values across all samples, allowing for an overall assessment of feature importance and the direction of their effects, whereas the waterfall plot visualizes, for each individual sample, how features sequentially contribute to the deviation from the baseline prediction. The analysis results are presented in Figs. 4 and 5.

The SHAP beeswarm plot analysis demonstrated that ‘Night Sleep Time’ consistently emerged as the most influential variable in XGBoost and LightGBM models, whereas ‘Day Sleep Time’ was identified as the most significant predictor in the first model. The contribution of ‘Night Sleep Time’ to the prediction was particularly prominent near its median range, as indicated by the dense concentration of SHAP values around the central axis. This observation suggests that maintaining a moderate duration of night sleep may be associated with a reduced risk of depression. Additional features such as ‘Age’, ‘Day Sleep Time’, ‘Religion Activity’, ‘Income Responsibility’, and ‘Diabetes’ were repeatedly highlighted as key predictors across all three models. A reduction in ‘Night Sleep Time’ generally corresponded to an increased likelihood of depressive outcomes, as inferred from the directionality of SHAP values. Conversely, active engagement in social behaviors, including participation in community or social groups such as ‘Social Club’ activities, appeared to mitigate predicted depression risks, underscoring the potential psychological benefits of social connectedness. The convergence of feature importance across ensemble models reinforces the interpretability and consistency of these predictors in relation to depressive symptomatology derived from behavioral and demographic input variables.

The waterfall plot analysis provided additional validation of feature contributions at the individual level. In all three models, ‘Night Sleep Time’ consistently exerted the strongest negative impact on the model output, significantly lowering the predicted probability of depression. This reinforces its role as a protective factor, suggesting that maintaining sufficient night sleep duration may help mitigate depressive risk. Other features such as ‘ReligionActivity’, ‘IncomeResponsibility’, and ‘Night Deep Time’ also contributed to reduced prediction scores, indicating their potential positive influence on mental health. In contrast, variables including Step activity, Diabetes, and Stroke were associated with an upward shift in the prediction score, implying that lower physical activity and the presence of chronic medical conditions may increase the likelihood of depression. These results emphasize the consistent explanatory strength of sleep related features, along with behavioral and clinical indicators, in shaping individualized depression risk estimations across multiple models.

### LIME Analysis

LIME (Local Interpretable Model-agnostic Explanations) is a representative XAI technique designed to provide local interpretability of machine learning model predictions. For a complex and nonlinear model *f*, LIME approximates the prediction for a specific input sample *x* using a simpler, interpretable model *g*. This objective can be expressed as follows in Eq. (3) where *L(f*,* g*,*π*_*x*_*)* represents the loss function that measures the discrepancy between the predictions of the original model *f* and the interpretable model *g* under a locality distribution *π*_*x*_​ around the sample *x*, and *Ω(g)* denotes a penalty term for the complexity of the surrogate model *g*.

3 $$\underset{g\in G}{argmin\;}\mathcal L(f,g,{\mathrm\pi}_{\mathrm x})+\Omega(g)$$

Through this optimization process, LIME constructs a simple linear model or decision tree that explains the major features contributing to the specific prediction outcome. In this study, LIME was used in parallel with SHAP, with particular attention given to LIME’s ability to provide local explanations. By training a simple model centered around each sample, LIME enables the intuitive identification of key features that most significantly influenced each prediction. This approach allowed us to examine the differences in feature contributions between individuals predicted as depressed and those classified as controls, thereby enhancing the interpretability and trustworthiness of predictions made by the complex model.

**Fig. 6:**
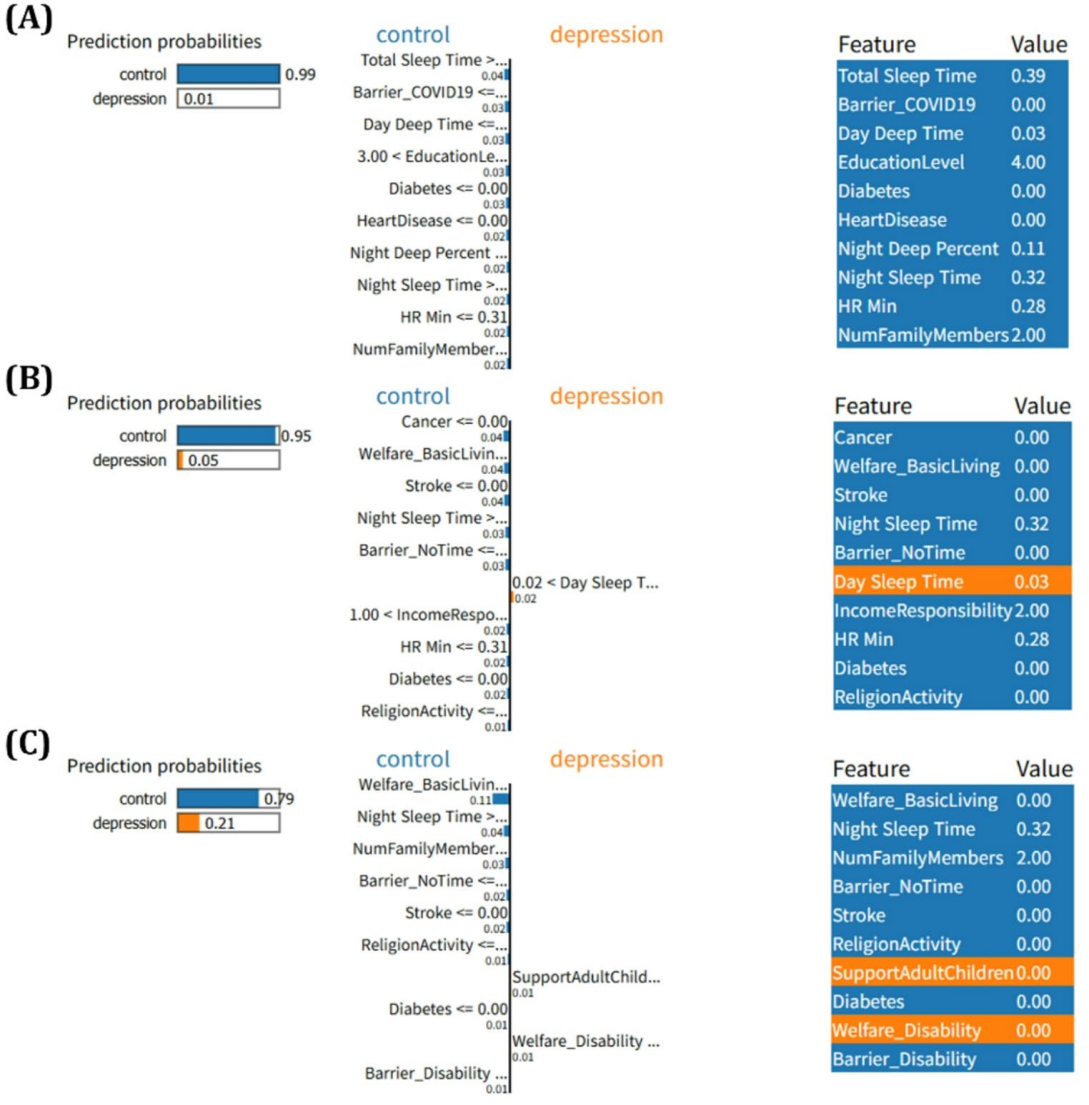
Local explanations of depression prediction using LIME across three ensemble models: (**A**) Random Forest, (**B**) XGBoost, and (**C**) LightGBM. Each plot illustrates the feature contributions for a selected individual, with bars representing the extent to which each feature increased or decreased the predicted probability of depression. The visualizations highlight how specific features locally influenced the model’s decision for each sample

Based on the LIME analysis results presented in Fig. 6, we further examined the key features that influenced prediction outcomes at the individual level. Among the participants classified as control, features such as ‘Night Sleep Time’, ‘Total Sleep Time’, and ‘HR Min’ were consistently associated with predictions toward the non-depressive class. Particularly, individuals with sufficient night and total sleep durations showed markedly lower predicted probabilities of depression, aligning with trends identified in the global SHAP analysis. In contrast, for individuals with higher predicted probabilities of depression, features such as ‘Welfare_BasicLiving’, ‘Stroke’, ‘Diabetes’, and ‘Barrier_NoTime’ contributed positively to the model output. Moreover, additional indicators including ‘Support for Adult Children’ and ‘Welfare_Disability’ appeared as influential factors in the third case, reflecting socioeconomic and caregiving related stressors. These findings suggest that while sleep-related features serve as protective factors at the individual level, the presence of chronic illness, limited time resources, and socioeconomic burden emerge as key drivers that elevate the predicted risk of depression.

### Feature Analysis

**Fig. 7:**
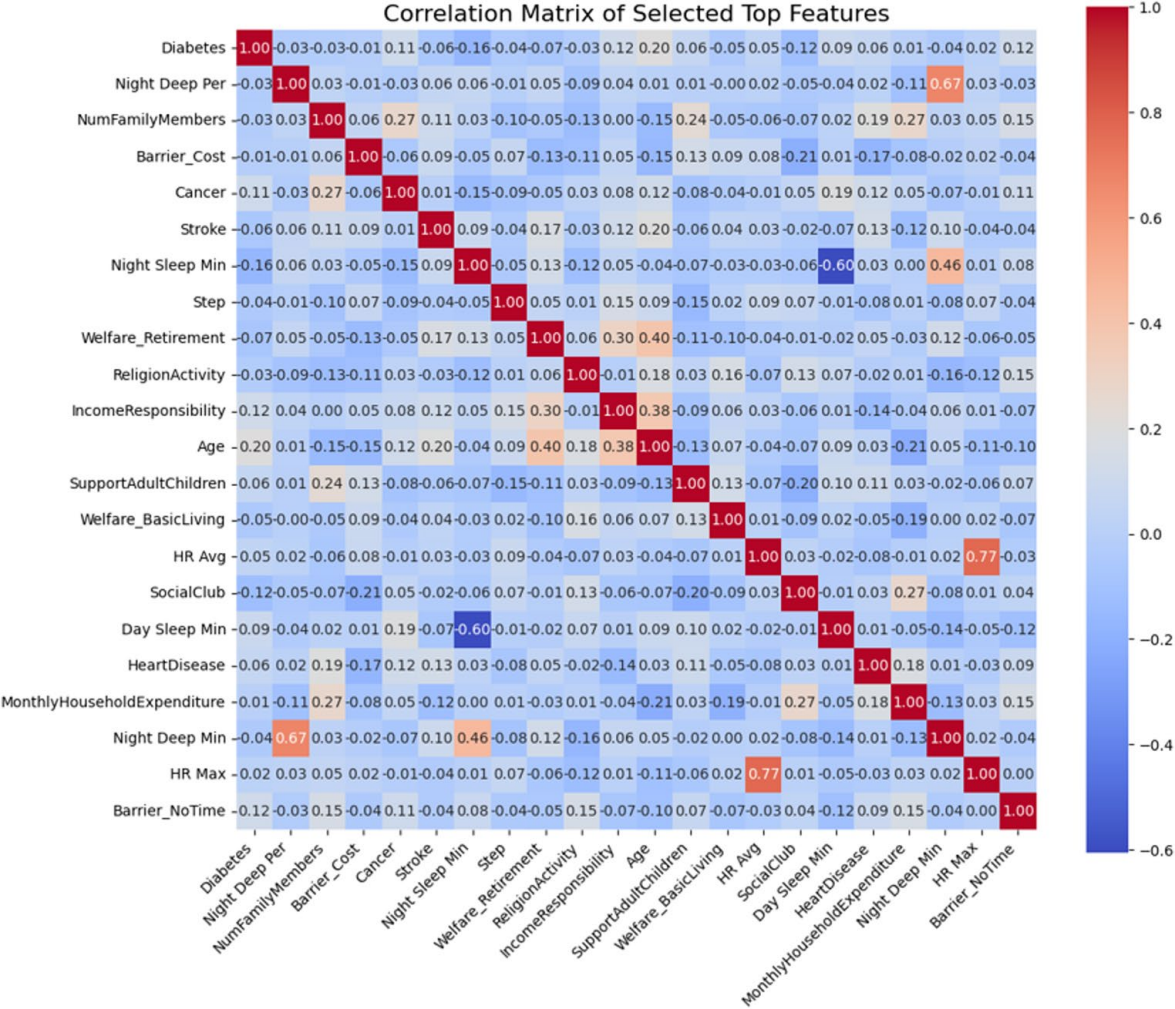
Correlation matrix of the unified top features selected from Random Forest, XGBoost, and LightGBM models

To evaluate the contribution of individual features to model predictions, feature importance analysis was performed using both SHAP and LIME methods. Based on the resulting importance scores, top-ranked features were selected for subsequent model training. To further assess potential redundancy among the selected features, a correlation matrix analysis was conducted on the integrated feature set, as shown in Fig. 7. The analysis revealed that most feature pairs exhibited low correlation coefficients, with the maximum absolute correlation observed at approximately 0.77. These results suggest that the selected features primarily provide independent information, thereby supporting the robustness of the subsequent deep learning model training based on the selected feature sets.

### Evaluation

Through the preceding analysis, key features were extracted from the three ensemble models previously described Random Forest, XGBoost, LightGBM. In this study, the top 15 important features identified from each model were selected to systematically evaluate the impact of feature selection on the performance of hybrid deep learning models. The hybrid models employed for evaluation were the 1D-CNN and MLP, each integrated with LightGBM, as previously outlined. Two experimental conditions were compared by training models either with the full set of features or exclusively with the selected important features. Model performance was evaluated using standard classification metrics, namely accuracy, precision, recall, F1-score, and area under the ROC curve (AUC), as defined by Eqs. (4), (5), (6), (7), and (8) respectively.

4 $$Accuracy=\frac{TP+TN}{TP+FP+FN+TN}$$

5 $$Precision=\frac{TP}{TP+FP}$$

6 $$Recall=\frac{TP}{TP+FN}$$

7 $$F1-score=2\times\frac{Precision\times Recall}{Precision+Recall}$$

8 $$AUC=\int_0^1TPR(x)\;dx$$

**Fig. 8:**
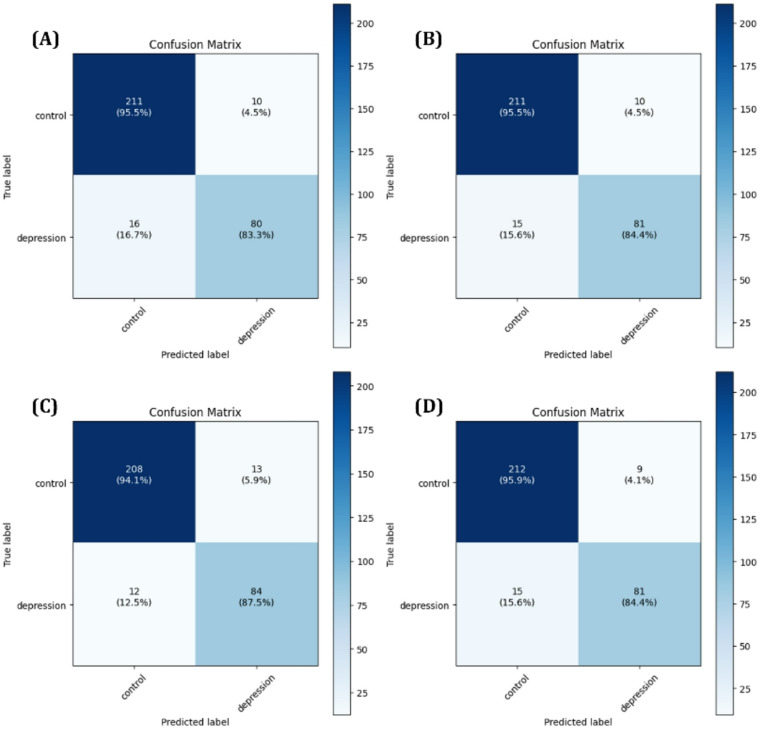
Confusion matrices for the 1D-CNN + LightGBM model: (**A**) using all features, (**B**) using features selected by Random Forest, (**C**) using features selected by XGBoost, and (**D**) using features selected by LightGBM

**Fig. 9:**
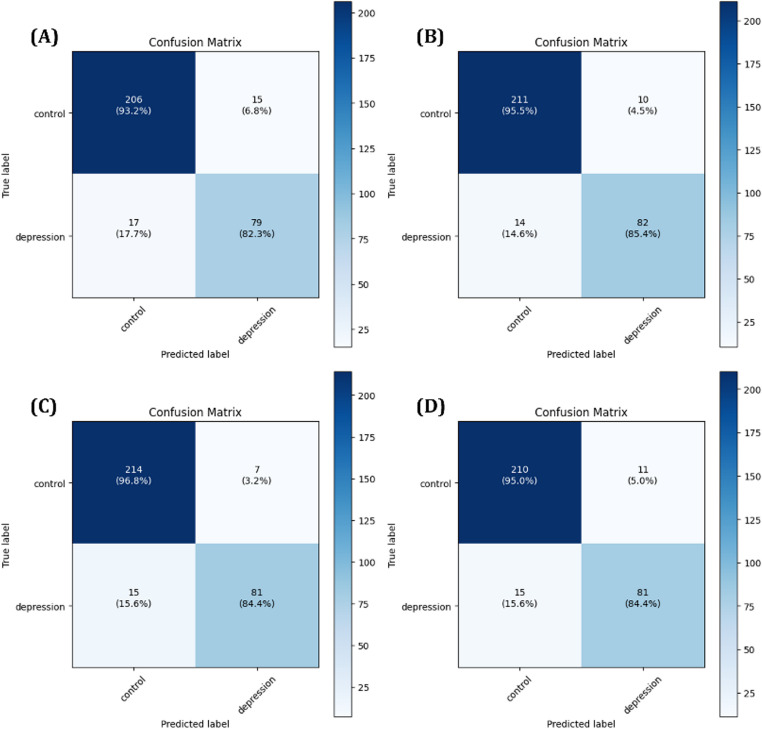
Confusion matrices for the MLP + LightGBM model: (**A**) using all features, (**B**) using features selected by Random Forest, (**C**) using features selected by XGBoost, and (**D**) using features selected by LightGBM

**Fig. 10:**
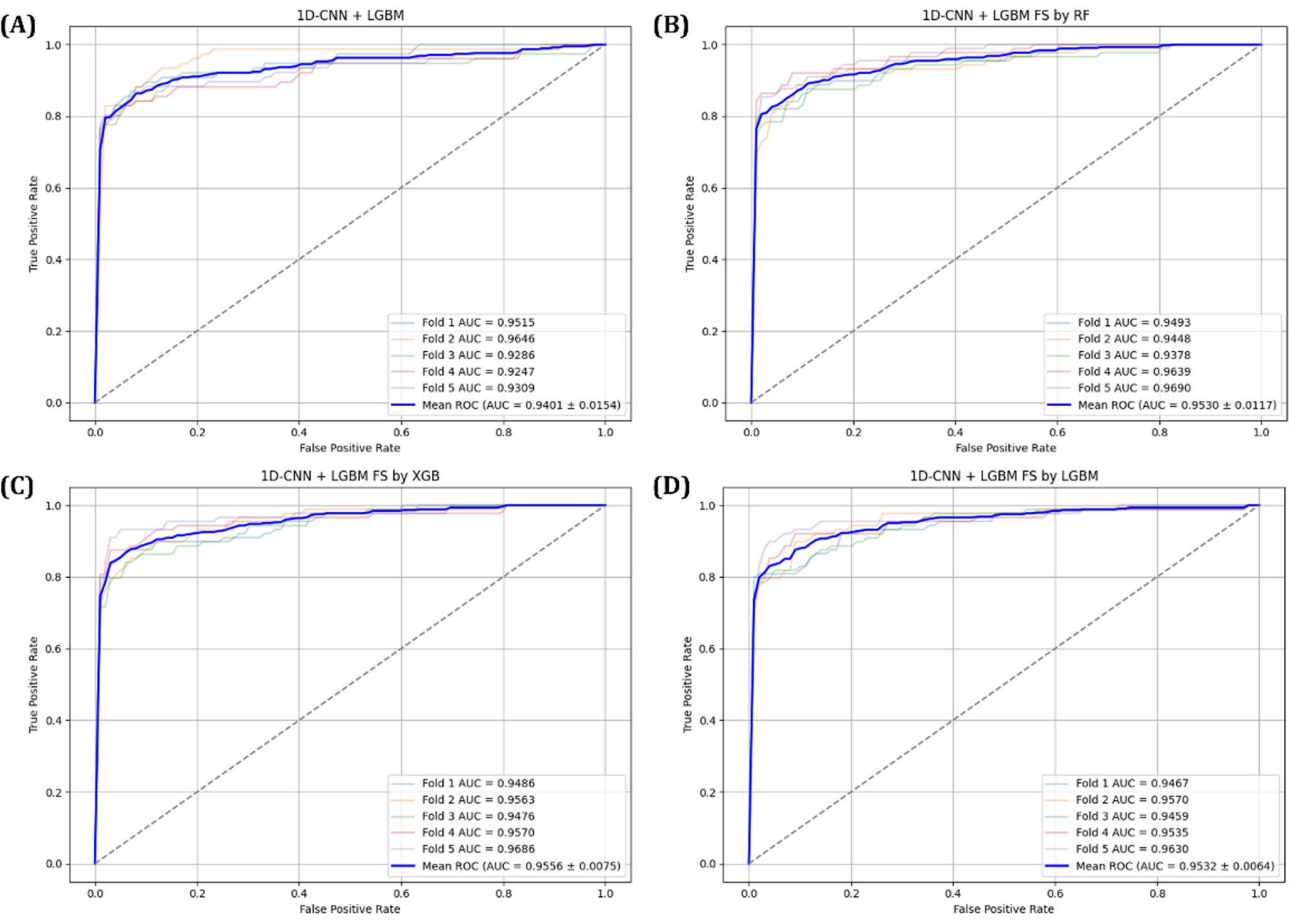
ROC curves for the 1D-CNN + LightGBM model: (**A**) using all features, (**B**) using features selected by Random Forest, (**C**) using features selected by XGBoost, and (**D**) using features selected by LightGBM

**Fig. 11:**
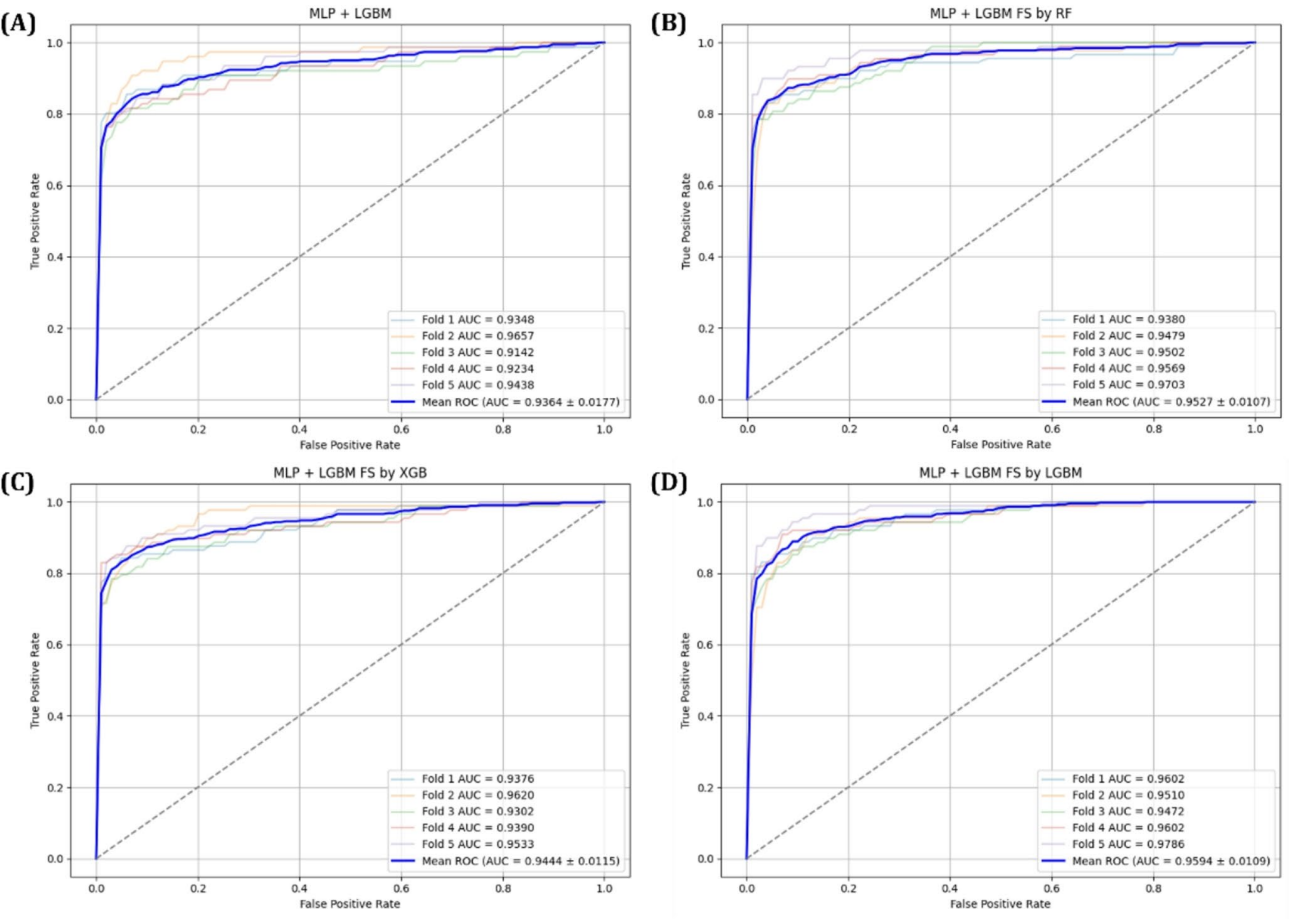
ROC curves for the MLP + LightGBM model: (**A**) using all features, (**B**) using features selected by Random Forest, (**C**) using features selected by XGBoost, and (**D**) using features selected by LightGBM

The predictive performance of each model was first assessed using confusion matrices, with results for the 1D-CNN+LightGBM model shown in Fig. 8 and for the MLP + LightGBM model in Fig. 9. In addition, ROC curves and AUC scores were analyzed during 5-Fold cross validation to evaluate the variability and generalization performance of the models, as shown in Figs. 10 and 11. A comprehensive summary of the quantitative results for all experimental conditions was provided Table 6, enabling an integrated comparison of the model’s classification performance. The experimental results confirmed that models trained using selected important features consistently outperformed those trained with all available features. Particularly, MLP + LightGBM hybrid model, trained with features selected via XGBoost, achieved the highest classification accuracy 93.43%. These findings empirically demonstrate that effective feature selection can significantly enhance model predictive performance, while simultaneously improving learning efficiency and model interpretability.

**Table 6:** Performance Comparison of Hybrid Models (1D-CNN+LGBM and MLP+LGBM) Using All Features and Top 15 Features Selected by Random Forest, XGBoost, and LightGBM

Models	Features	Accuracy	Precision	Recall	F1-Score	AUC
1D-CNN + LGBM	All	0.9180	0.8889	0.8333	0.8602	0.9401
	FS - RF	0.9211	0.8901	0.8438	0.8663	0.9530
	FS - XGB	0.9211	0.8660	0.8750	0.8705	0.9556
	FS - LGBM	0.9243	0.9000	0.8438	0.8710	0.9532
MLP + LGBM	All	0.8991	0.8404	0.8229	0.8316	0.9364
	FS - RF	0.9243	0.8913	0.8542	0.8723	0.9527
	FS -XGB	0.9306	0.9205	0.8438	0.8804	0.9444
	FS - LGBM	0.9180	0.8804	0.8438	0.8617	0.9594

## Discussion

In this study, a hybrid deep learning and machine learning model was designed and evaluated to predict depressive symptoms using tabular data collected from wearable devices. Specifically, SHAP and LIME were employed to identify and interpret key features contributing to model predictions. A central focus of this work was on feature importance analysis, which not only provided insights into the most influential predictors but also enabled a clearer understanding of their directionality and individual-level variability.

The SHAP analysis revealed that ‘Night Sleep Time’, ‘Day Sleep Time’, and ‘Religion Activity’ consistently emerged as high importance features across all ensemble models. Among them, Night Sleep Time demonstrated a robust and stable protective effect, with durations close to the median being associated with a lower risk of depression. In addition, socioeconomic variables such as ‘Income Responsibility’ and ‘Barrier_NoTime’ were found to have considerable impact, suggesting that daily life constraints and social obligations may play a meaningful role in mental health outcomes. Further validation using waterfall plots confirmed these findings at the individual level. Features such as ‘Night Sleep Time’, ‘Religion Activity’, and ‘Night Deep Time’ contributed to lowering the predicted depression risk, while lower step activity, the presence of stroke, and diabetes increased the likelihood of depression. These trends were mirrored in the LIME analysis, which consistently highlighted features that contributed to non-depression predictions, including Total Sleep Time and minimum heart rate, as well as those associated with higher depression risk, such as ‘Welfare_BasicLiving’, ‘SupportAdultChildren’, and ‘Welfare_Disability’. These observations underscore the importance of personalized interpretation in mental health prediction systems. The overall feature patterns revealed by the XAI analysis are not only statistically meaningful but also contextually relevant, reflecting diverse physiological, behavioral, and socioeconomic influences that could serve as potential biomarkers for depression. Correlation matrix analysis further supported the independence of these features, with the highest absolute correlation coefficient remaining around 0.77, which improved the stability of the model training after feature selection.

Building on this refined set of features, two hybrid models were developed by combining deep learning structures, namely 1D convolutional neural networks and multilayer perceptrons, with the LightGBM algorithm. These models, when trained on the selected features, consistently outperformed models using all available features across multiple evaluation metrics, including accuracy, precision, recall, F1-score, and AUC. Among them, the MLP integrated with LightGBM using XGBoost-based feature selection achieved the highest classification accuracy of 93.43%.

Taken together, these results suggest that combining interpretable feature selection with hybrid modeling not only enhances prediction accuracy but also improves the transparency and reliability of AI-assisted mental health assessments, paving the way for effective personalized early detection strategies for depression.

### Limitations

Despite the promising results obtained in this study, several limitations should be acknowledged. First, the dataset used for model development was derived from tabular data collected via wearable smartwatches and represented a relatively limited population cohort. Although the number of participants was adequate for initial model development, the data were collected from a single regional cohort and may reflect specific demographic or lifestyle characteristics. Consequently, the findings may not be generalizable to broader or more heterogeneous populations, including those with varying socio-cultural or clinical backgrounds. In addition, wearable device-based studies are inherently affected by participant compliance, as devices may not be worn consistently, particularly during sleep, and wearing patterns may differ according to symptom severity. To reduce the impact of non-wear periods or missing data, this study used monthly aggregated average physiological features rather than raw daily or continuous time-series signals. Samples were excluded only when monthly averages could not be reliably computed due to insufficient data, and no additional exclusion criteria based on daily wearing time were applied.

Nevertheless, differences in device-wearing behavior, especially among participants with more severe depressive symptoms, may not be fully captured by monthly aggregation alone. Such unmeasured variability in device compliance represents a limitation of the current study. Future research should consider incorporating explicit measures of wearing time or device usage patterns or treating compliance-related features as additional model inputs, to further improve data quality and model robustness. Second, although SHAP- and LIME-based feature selection improved model interpretability and predictive performance, the identified feature sets were derived within a specific dataset–model context. As a result, the relative importance and predictive contribution of these features may vary when applied to external datasets, alternative model architectures, or across different temporal contexts due to behavioral, environmental, or seasonal variations. To establish these features as stable and generalizable digital biomarkers for depression-related outcomes, further validation using independent cohorts and long-term longitudinal data is necessary.

Third, this study was limited to structured tabular data obtained from wearable devices. While such data provide valuable summaries of physiological and behavioral patterns, they exclude other potentially informative modalities such as raw sensor time-series signals (e.g., heart rate variability waveforms), contextual information, or high-resolution psychological assessments (e.g., ecological momentary assessment). In addition, although depressive symptoms severity represents an important clinical dimension, the dataset collected under real-world clinical and observational conditions contained a limited and imbalanced number of participants across different PHQ-9 severity groups. This constrained the statistical robustness required for reliable severity-specific modeling or stratified performance comparisons. Consequently, the proposed model was designed to focus on risk assessment and identification of elevated depressive symptoms rather than fine-grained severity classification.

Furthermore, depressive symptoms in this study were assessed using self-reported PHQ-9 questionnaires, which are subject to reporting bias, recall effects, and individual differences in symptoms perception. While PHQ-9 is widely used as a validated screening tool, it does not substitute for clinician administered diagnostic interviews. Additionally, the aggregation of wearable derived features at a monthly resolution may obscure short-term fluctuations in mood or symptoms dynamics, particularly around the onset or remission of depressive episodes. Future work should therefore explore multi-scale temporal modeling approaches, integrating daily or weekly sensor features with repeated psychological assessments and raw signal level representations.

Incorporating such multimodal data sources and larger, more balanced cohorts across symptoms severity levels could provide richer representations of individual mental health states and further improve the clinical utility, level of detail, and personalization of predictive models. Ultimately, these efforts may support the development of more comprehensive and real-world applicable AI-based screening and risk stratification tools for mental health monitoring.

## Data Availability

No datasets were generated or analysed during the current study.
